# Deep-submicron Graphene Field-Effect Transistors with State-of-Art *f*_*max*_

**DOI:** 10.1038/srep35717

**Published:** 2016-10-24

**Authors:** Hongming Lyu, Qi Lu, Jinbiao Liu, Xiaoming Wu, Jinyu Zhang, Junfeng Li, Jiebin Niu, Zhiping Yu, Huaqiang Wu, He Qian

**Affiliations:** 1 Institute of Microelectronics, Tsinghua University, Beijing, 100084, China; 2 Department of Electrical and Computer Engineering, Rice University, Houston, TX 77005, USA; 3 Institute of Microelectronics, Chinese Academy of Sciences, Beijing, 100029 China; 4 Tsinghua National Laboratory for Information Science and Technology (TNList), Beijing, 100084 China

## Abstract

In order to conquer the short-channel effects that limit conventional ultra-scale semiconductor devices, two-dimensional materials, as an option of ultimate thin channels, receive wide attention. Graphene, in particular, bears great expectations because of its supreme carrier mobility and saturation velocity. However, its main disadvantage, the lack of bandgap, has not been satisfactorily solved. As a result, maximum oscillation frequency (*f*_*max*_) which indicates transistors’ power amplification ability has been disappointing. Here, we present submicron field-effect transistors with specially designed low-resistance gate and excellent source/drain contact, and therefore significantly improved *f*_*max*_. The fabrication was assisted by the advanced 8-inch CMOS back-end-of-line technology. A 200-nm-gate-length GFET achieves *f*_*T*_/*f*_*max*_ = 35.4/50 GHz. All GFET samples with gate lengths ranging from 200 nm to 400 nm possess *f*_*max*_ 31–41% higher than *f*_*T*_, closely resembling Si n-channel MOSFETs at comparable technology nodes. These results re-strengthen the promise of graphene field-effect transistors in next generation semiconductor electronics.

In order to conquer short channel effects, two-dimensional materials receive wide attention nowadays^1^. Graphene, in particular, bears great expectations because of its supreme carrier transport properties^2^. However, even though the cutoff frequency (*f*_*T*_) of graphene field-effect transistors (GFETs) exceed Si transistors^3^,^4^, the maximum oscillation frequency (*f*_*max*_), as the most relevant metric to circuit performance, seriously lags behind. Promising circuit applications are limited in passive circuits^5^,^6^,^7^,^8^,^9^. Typical *f*_*max*_ values are one order of magnitude lower than *f*_*T*_^3^,^10^,^11^,^12^,^13^. For instance, the 46-nm-gate-length GFET in Cheng *et al*.’s work delivered *f*_*T*_/*f*_*max*_ of 212/8 GHz^3^. Wu *et al*. achieved *f*_*T*_/*f*_*max*_ = 300/30 GHz and *f*_*T*_/*f*_*max*_ = 120/44 GHz for GFETs with channel lengths of 40 nm and 120 nm, respectively^10^. The absence of bandgap is the primary drawback that limits *f*_*max*_^1^. Introducing a bandgap by using bilayer or multilayer graphene is a straightforward idea. However, it suffers from synthesis difficulties^14^,^15^. On the other hand, reduction of parasitic effects is a practical solution^16^,^17^,^18^. Parasitic effects such as overlapping capacitance, contact resistance and gate resistance influence transistors’ RF performance. Several groups have already increased GFETs’ *f*_*max*_ by lowering the gate resistance^16^,^17^,^18^,^19^. Heer *et al*. achieved *f*_*max*_ up to 70 GHz for a 100 nm gate device, using mushroom T-shape top gates to lower the gate resistance^16^. Han *et al*. employed T-shape buried gates. Their work delivered *f*_*max*_ 25–43% higher than *f*_*T*_, with the highest *f*_*max*_ of 20 GHz^17^.

In this work, the modern CMOS back-end-of-line (BEOL) technology has been employed to fabricate deep-submicron GFETs. GFETs with gate lengths ranging from 100 nm to 400 nm have been fabricated on 200 mm wafers by recently reported passive-first-active-last inverted process^8^,^9^,^20^. In particular, buried gates with depth-to-width ratio up to six folds were achieved for the purpose of lowering the gate resistance. These GFETs achieve RF metrics (i.e. *f*_*max*_ and *f*_*T*_) close to Si n-channel MOSFETs at comparable technology nodes. In particular, the 200-nm-gate-length GFET generates *f*_*max*_*/f*_*T*_* = 50/35.4* *GHz*. The *f*_*max*_/*f*_*T*_ ratio is also among the highest in literature.

## Results and Disscussion

### Fabrication

**Figure 1:**
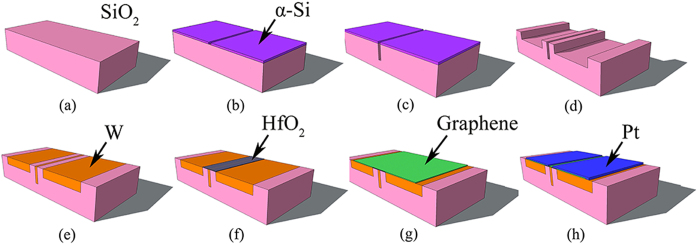
Process flow. (**a**) High-res Si substrate. (**b**) Definition of the gate on hard mask sacrificing layer. (**c**) Etching of the gate. (**d**) Definition and etching of the source/drain region bottom contacts. (**e**) W deposition and CMP. (**f**) Deposition of HfO_2_ gate dielectric. (**g**) Graphene transfer. (**h**) Patterning source/drain top contacts by lift-off process.

Schematic of the fabrication process flow is demonstrated in Fig. 1(a–h). 200 mm Si wafers with high resistivity (>1000 Ω cm) were used for the purpose of reducing substrate losses. 5 μm thick SiO_2_ layer was first deposited by PECVD method as the insulator layer. Metal structures were formed by Damascene process. For the purpose of increasing the depth-to-width ratio of the buried gates, 100 nm thick α-Si was deposited as the hard mark sacrifice layer. Definition of the deep-submicron gates was enabled by electron-beam lithography (EBL). Reactive ion etching (RIE) was used to realize the trenches. Owing to the large selection ratio between SiO_2_ and α-Si, the depth-to-width ratio was significant increased up to six, as illustrated in Fig. 1(b,c). After removal of α-Si, source/drain bottom contacts, interconnects and probing pads were defined by stepper lithography and etching. Bottom metals serve as an addition to top metals to ensure good contacts^21^. Then, tungsten was deposited by CVD method to fill in the trenches, followed by chemical-mechanical-planarization (CMP). Besides removing extra tungsten, it also guarantees the flatness of the wafer, necessary for the successfulness of the following graphene transfer process. HfO_2_ with equivalent oxide thickness (EOT) of 2 nm was deposited by atomic layer deposition (ALD) as the gate dielectric. The relative dielectric constant was about 20. The dielectric was defined by stepper lithography and removed by inductively coupled plasma (ICP) etching with BCl_3_ source.

**Figure 2:**
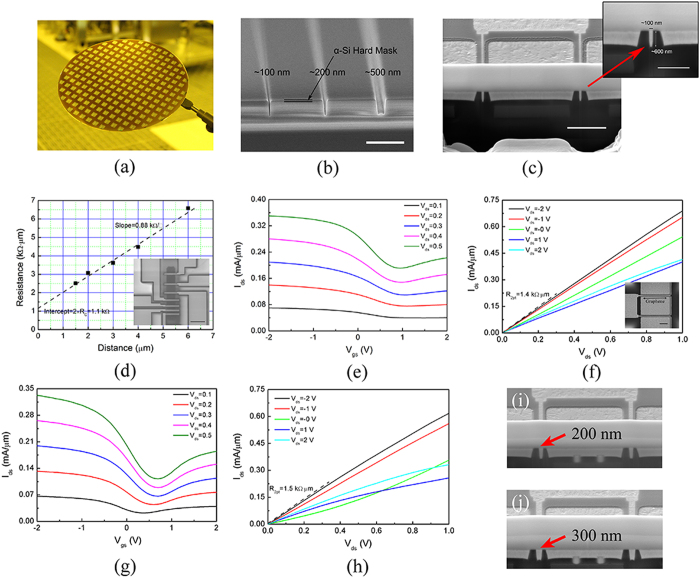
DC characterization. (**a**) A fabricated 200 mm Si wafer. (**b**) Deep-submicron buried gate trenches etched with α-Si mask. Scale bar: 2 μm. (**c**) Cross-section view of a 100-nm-gate-length GFET structure. Scale bar: 2 μm. The inset shows the close-up view of the 100 nm buried gate. Scale bar: 1 μm. (**d**) TLM measurement of graphene-Pt contact resistance, which results in *R*_*C*_ = 550 Ω μm. The inset shows the SEM image of the TLM device. Scale bar: 10 μm. Transfer (**e**) and output (**f**) characteristics of the 100-nm-gate-length GFET. Transfer (**g**) and output (**h**) characteristics of a 300-nm-gate-length GFET. Cross-section views of the structures of a 200-nm-gate-length GFET (**i**) and a 300-nm-gate-length GFET (**j**).

Figure 2(a) shows a photograph of a fabricated 200-mm wafer. Stand-alone submicron gate trenches with widths of 100 nm, 200 nm and 500 nm are displayed in Fig. 2(b). α-Si hard mask about 100 nm thick was indicated. Cross-section view of a 100-nm-gate-length GFET structure is demonstrated in the inset of Fig. 2(c), with the gate trench 100 nm long and 600 nm deep. Graphene in this work was formed by CVD method on Pt foils as previously reported^22^,^23^. “Bubbling” method was used to transfer graphene to the patterned wafer on a die-by-die basis, limited by the maximum size of Pt foil^22^,^23^. Graphene channels were patterned by contact mode contact lithography and oxygen plasma etching. All GFETs employed two-finger layout with each finger 6 μm wide. The source/drain contacts were defined by EBL, and went through 5 min ultraviolet-ozone (UVO) treatment before sputtering of 40 nm Pt and the following lift-off process. The ungated source/drain-gate spacer was about 200 nm. Stand-alone Hall device with size 36 μm × 8 μm resulted in carrier mobility about 3400 cm^2^v^−1^-s^−1^. Higher mobility is expected in fabricated GFETs, as smaller areas are less prone to defects. The probing pads feature 100 μm pitch in ground-signal-ground (GSG) layout. The GFETs have not gone through the passivation step for convenience. Various dielectrics passivation layers, such as Si_3_N_4_^24^, BN^25^, Al_2_O_3_^26^, etc., can be considered to further increase the stability and reliability of the graphene devices.

Stand-alone transfer length measurement (TLM) patterns were fabricated along with the GFETs to measure the contact resistance, *R*_*C*_. It indicates *R*_*C*_ of 550 Ω um as well as the sheet resistance of 880 Ω/sqr, as shown in Fig. 2(d) with the inset showing the SEM image of the TLM pattern. Typical metal contact resistance for CVD graphene ranges from few hundreds Ω to few kΩ^27^,^28^,^29^,^30^. *R*_*C*_ in this work is among the lowest, attributed to the high work function of Pt that induces more carriers underneath and UVO treatment that enhances the binding between metal and graphene^30^,^31^. Other metal deposition recipes may also be considered as sputtering on graphene causes certain disorders. Figure 2(e) shows the transfer characteristics of the 100-nm-gate-length GFET with on-off ratio of roughly 2 folds. Figure 2(f) displays the output characteristics. It is worth noting that the two-point resistance R_2pt_ is 1.4 kΩμm, indicating the contact resistance is less than 700 Ωμm, consistent with the stand-alone TML measurement. Top view of a fully processed 100-nm-gate-length GFET is shown in the inset of Fig. 2(f). Figure 2(f,g) present the transfer and output characteristics of a 300-nm-gate-length GFET, respectively. As expected, it demonstrates stronger gate modulation (i.e. transconductance) compared to the 100 nm counterpart. The two-point resistance R_2pt_ also slightly increases to 1.5 kΩμm as the drain-to-source distance becomes longer. Figure 2(i,j) show the cross-section views of 200- and 300-nm-gate-length GFETs, respectively.

### RF Performance and Discussion

**Figure 3:**
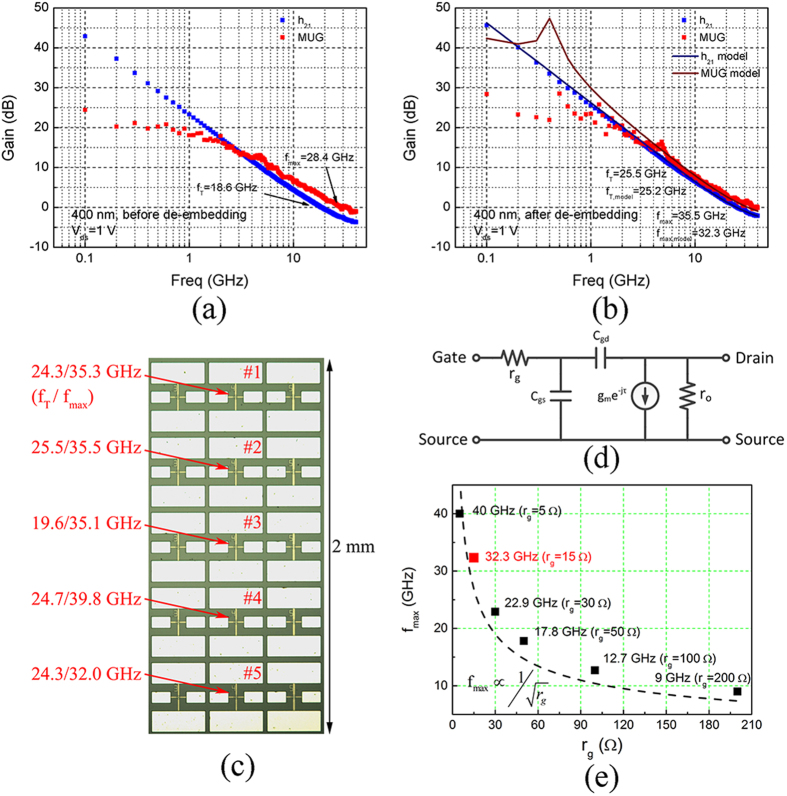
RF characterization. (**a**) h_21_ and MUG of a 400-nm-gate-length GFET before de-embedding. (**b**) h_21_ and MUG of the 400-nm-gate-length GFET after de-embedding and small-signal model fitting. (**c**) *f*_*T*_/*f*_*max*_ of five 400-nm-gate-length GFETs across an array. (**d**) Equivalent circuit of the small-signal model. (**e**) *f*_*max*_’s depedence on gate resistance, *r*_*g*_.

High-frequency S-parameters of the GFETs were measured up to 40 GHz under ambient atmosphere using Agilent N8230C network analyzer. The system was calibrated with short-open-load-through (SOLT) method. Three-step de-embedding procedure was used^32^, which employed “open”, “through” and “short” structures to de-embed on-wafer parasitic components. (See Supporting Information) h_21_ and MUG of a 400-nm-gate-length GFET are displayed in Fig. 3(a,b). It delivers *f*_*T*_/*f*_*max*_ of 18.6/28.4 GHz before de-embedding, and *f*_*T*_/*f*_*max*_ of 25.5/35.5 GHz after de-embedding. As a reference, a recently reported 450-nm-gate-length GFET delivered *f*_*T*_/*f*_*max*_ of 11.5/15 GHz^17^. Five successive GFETs with 400 nm gate length are shown in Fig. 3(c), which indicate excellent performance uniformity. A small-signal model is built which enables the analysis on the role of each parameter. The schematic of the small-signal equivalent circuit is presented in Fig. 3(d). Different from that of a conventional transistor, *r*_*g*_ lies in the outer equivalent position on neither of gate-source (*C*_*gs*_) and gate-drain (*C*_*gd*_) capacitance branches. It is because GFETs could not effectively pinch off and their behavior resembles linear-region conventional transistors. Fitting of the 400-nm-gate-length GFET with the small-signal model is demonstrated in Fig. 3(b), which delivers a close *f*_*T*_/*f*_*max*_ *of* 25.2/32.3 GHz. Values for each component of the small-signal model are displayed in Table 1. The raw values extracted from the measured S-parameters are shown in Supporting Information. They are relatively stable in the measurement frequency range, confirming the effectiveness of the small-signal model.

To verify *r*_*g*_’s role in *f*_*max*_, we varied *r*_*g*_ in a range including 5 Ω, 15 Ω, 30 Ω, 50 Ω, 100 Ω and 200 Ω, as depicted in Fig. 3(e). *r*_*g*_ = 15 Ω, marked red, generates the closest fitting as shown in Fig. 3(b). *f*_*max*_ increases or decreases with lower or higher *r*_*g*_, respectively. *f*_*max*_ is inversely proportional to the square root of *r*_*g*_, as indicated by the dashed guideline in Fig. 3(e), which is consistent with theoretical derivation as follows. (Derivation in detail is shown in Method section).


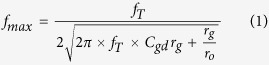


Unmodified gate is the primary reason why ordinary GFETs usually generate *f*_*max*_ one to two orders of magnitude lower than *f*_*T*_ 3,10,11,12,13. Unlike *f*_*max*_, *f*_*T*_ is independent of *r*_*g*_. It is worth mentioning that *R*_*s*_ and *R*_*d*_ have already been included in the transconductance term, *g*_*m*_, of the small-signal model. Larger contact resistance leads to smaller *g*_*m*_, thus *f*_*T*_ and *f*_*max*_. The influence of *R*_*s*_ and *R*_*d*_ on *g*_*m*_ can be addressed by physics-based large-signal compact models^33^.

**Figure 4:**
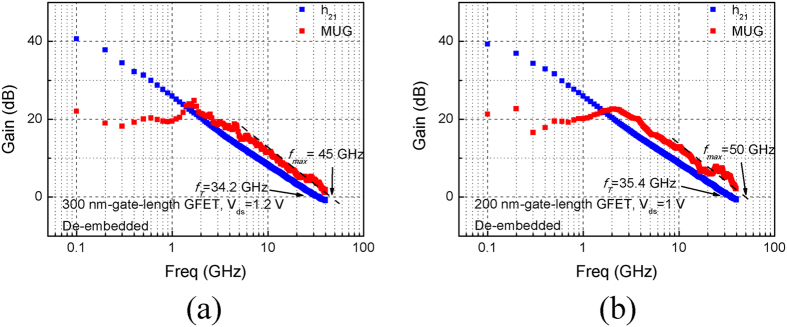
RF performance of the de-embeded 300- and 200-nm-gate-length GFETs. h_21_ and MUG of a 300-nm-gate-length GFET (**a**) and a 200-nm-gate-length GFET (**b**).

The h_21_ and MUG of a de-embedded 300-nm-gate-length GFET biased at V_ds_ = 1.2 V is shown in Fig. 4(a). It achieves *f*_*T*_/*f*_*ma*x_ = 34.2/45 GHz. Before de-embedding, *f*_*T*_ and *f*_*ma*x_ are 21.6 and 40 GHz, respectively (Supporting Information). The h_21_ and MUG of a de-embedded 200-nm-gate-length GFET biased at V_ds_ = 1.0 V is shown in Fig. 4(b). *f*_*T*_/*f*_*ma*x_ equal 35.4/50 GHz. Prior to de-embedding, *f*_*T*_ and *f*_*ma*x_ are 21.3 and 42 GHz, respectively (Supporting Information). The dash guide lines in these figures are in ideal −20 dB/dec slope for extrapolating *f*_*max*_^34^. The *f*_*max*_ values of the 200-, 300- and 400-nm-gate-length GFETs outperform previous works with comparable gate lengths to the best of our knowledge. As parasite effect plays a larger role with shorter gate length, the 100-nm-gate-length GFET is retained for more careful characterization in following works. *f*_*T*_’s dependence on gate length is shown in Supporting Information, revealing a relationship near to 1/L. Limited discrepancy may result from source/drain contact resistances, which play an unignorable role in GFETs^33^.

**Figure 5:**
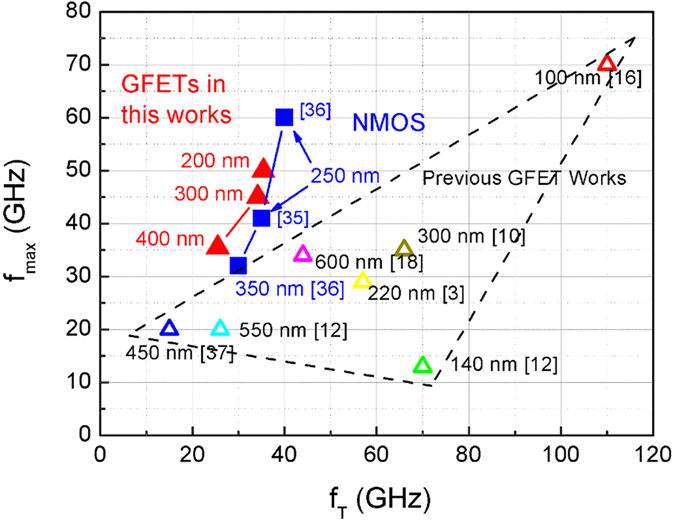
Comparing *f*_*T*_ and *f*_*max*_ of published GFETs, Si n-channel MOSFETs and GFETs in this work. Solid blue squares: Si n-channel MOSFETs at 0.25 μm^35^,^36^ and 0.35 μm^36^ technology nodes. Solid red triangles: GFETs in this work. Hollow triangles: published submicron GFETs^3^,^10^,^12^,^16^,^18^,^37^.

A comparison with recently published GFETs and Si n-channel MOSFETs (NMOSs) with similar gate lengths is shown in Fig. 5. *f*_*T*_/*f*_*max*_ of the GFETs is close to that of typical NMOSs at 0.25 μm and 0.35 μm technology nodes. And the *f*_*max*_/*f*_*T*_ ratio largely exceeds previous GFET works.

In summary, the advanced CMOS BEOL process has been employed in deep-submicron GFET fabrication. Thanks to the well-designed buried gate structure and lowered contact resistance, *f*_*max*_ has been increased significantly higher than before, rivaling Si transistor at comparable technology nodes. Considering its 8-inch wafer standard-process fabrication, GFETs are nearer to mass-production than ever. With the inverted process flow, one can also envision that future graphene RF components could be realized on CMOS backbones.

## Methods

### Graphene Synthesis and Transfer

Large scale monolayer graphene films were grown on 180 μm thick Pt foils (99.9 wt % metal basis, 10 mm × 10 mm) under ambient-pressure chemical vapor deposition (APCVD) method. The temperature was 1000 °C and CH_4_/H_2_ flow rate was set as 4.5/500 sccm. After growth, Pt foils are quickly pulled out of the high temperature area. PMMA photoresist was spun on graphene/Pt foil as the scaffold. Electrochemical delamination in NaOH solution was used to peel the graphene/PMMA off and transfer to pre-patterned dies. Raman spectrum of the monolayer graphene is shown in Supporting Information, Fig. S1.

### Derivation of *f*_*max*_-*r*_*g*_ relationship

**Figure 6:**
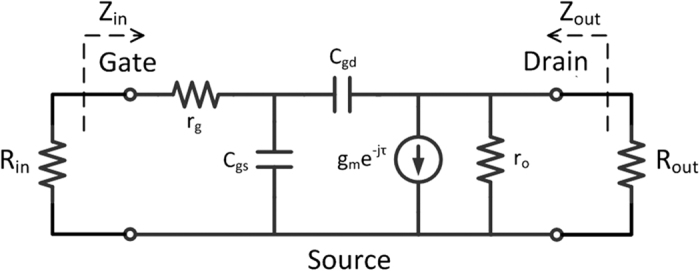


The equivalent circuit of the small-signal model is shown in Fig. 6. Firstly, the input impedance, *Z*_*in*_, and output impedance, *Z*_*out*_, are calculated:


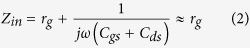



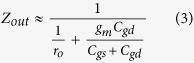


The input and output ports have to be conjugate-matched for maximum power transfer. Therefore, we have *R*_*in*_ = *Z*_*in*_ and *R*_*out*_ = *Z*_*out*_. Then, MUG can be expressed as


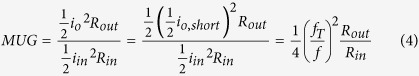


When MUG = 1,


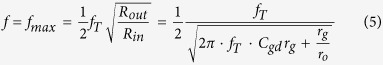


## Additional Information

**How to cite this article**: Lyu, H. *et al*. Deep-submicron Graphene Field-Effect Transistors with State-of-Art *f*_max_. *Sci. Rep.* **6**, 35717; doi: 10.1038/srep35717 (2016).

## Supplementary Material

Supplementary Information

## Figures and Tables

**Table 1:** Values of the small-signal model parameters.

r_g_ (Ω)	C_gs_ (fF)	C_gd_ (fF)	r_ds_ (Ω)	g_m_ (mS)	τ
15	17.5	26	117	5.6	3.12
