# Supplementary material for: Deep-submicron Graphene Field-Effect Transistors with State-of-Art fmax
Source: Sci Rep. 2016 Oct 24;6:35717. doi: 10.1038/srep35717 (PMC5075922; doi:10.1038/srep35717)
Supplement: Supplementary Information [file srep35717-s1.pdf]

Supporting Information

Deep-Submicron Graphene Field-Effect Transistors with State-of-Art f_{max}

Hongming Lyu[1,3,], Qi Lu[1], Jinbiao Liu[4], Xiaoming Wu[1], Jinyu Zhang[1], Junfeng Li[4], Jiebin Niu[4], Zhiping Yu[1], Huaqiang Wu[1,2,]*, and He Qian[1,2]*

[1]Institute of Microelectronics, Tsinghua University, Beijing, 100084, China

[2]Tsinghua National Laboratory for Information Science and Technology (TNList), Beijing, 100084, China,

[3]Department of Electrical and Computer Engineering, Rice University, Houston, TX 77005, USA

[4]Institute of Microelectronics, Chinese Academy of Sciences, Beijing, 100029, China

Corresponding authors:

* E-mail: hongming.lv@gmail.com

* E-mail: wuhq@tsinghua.edu.cn

Raman Spectrum of Graphene:

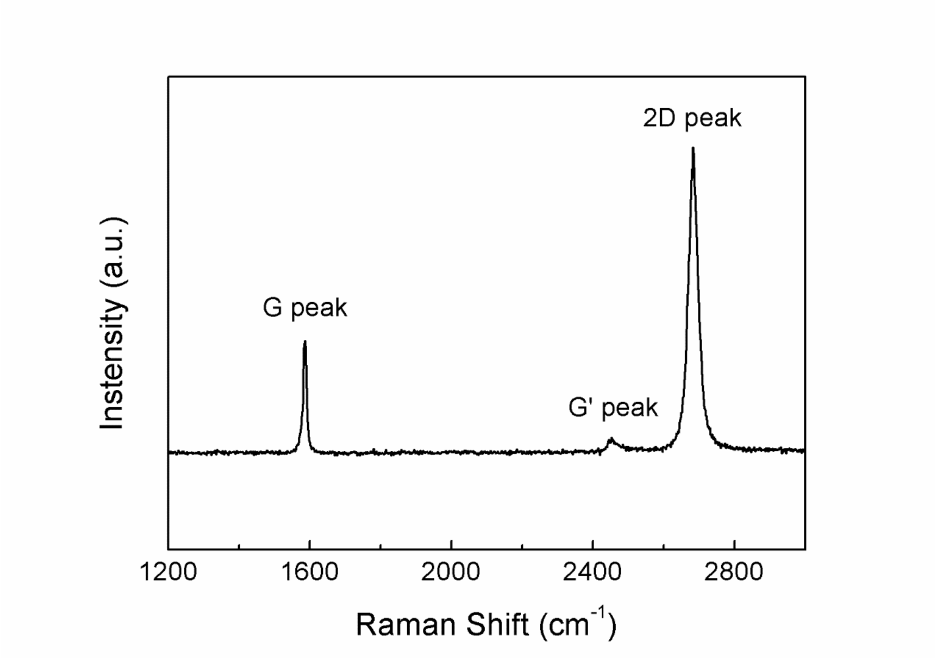

Figure S1. Raman spectrum of the graphene in this work.

Hall Device Measurement:

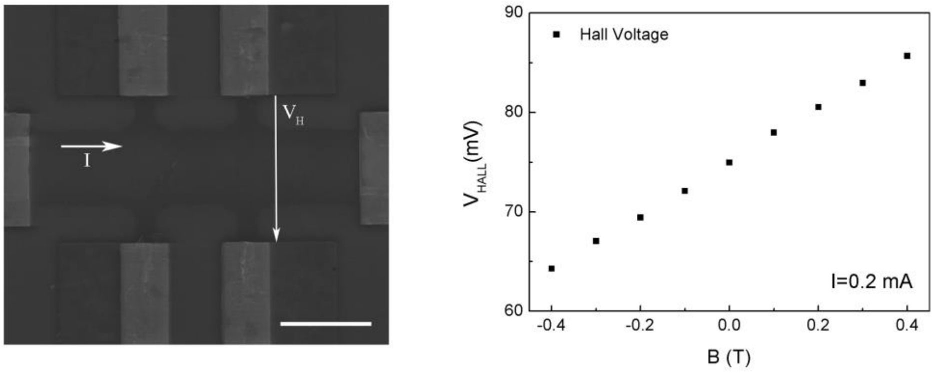

(a) (b)

Figure S2. The hall device measurement. (a) SEM image of the Hall device. The length and width of Hall bar are 36 μm and 8 μm, respectively. Scale bar: 10 μm. (b) Measured Hall voltage (V_{HALL}) vs. magnetic field (B).

The SEM image of the hall device is shown in Figure S2a. Figure S2b demonstrates the measured Hall voltage (V_{HALL}) vs. magnetic field (B). The Hall coefficient is defined as

$$R_H = \frac{V_H}{BI}$$

The conductance of Hall bar is $\sigma = 2\ mS$. The carrier mobility can be calculated as

$$\mu = R_H \cdot \sigma \approx 3400\, cm^2 / V \cdot s$$

De-embedding Structures and S-parameters:

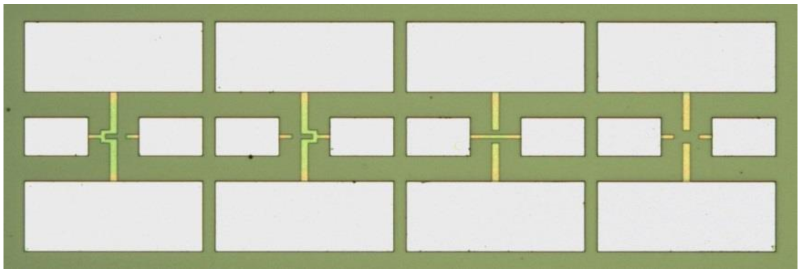

Figure S3. Optical image of de-embedding structures. From left to right: Short1, Short2, Thru, and Open.

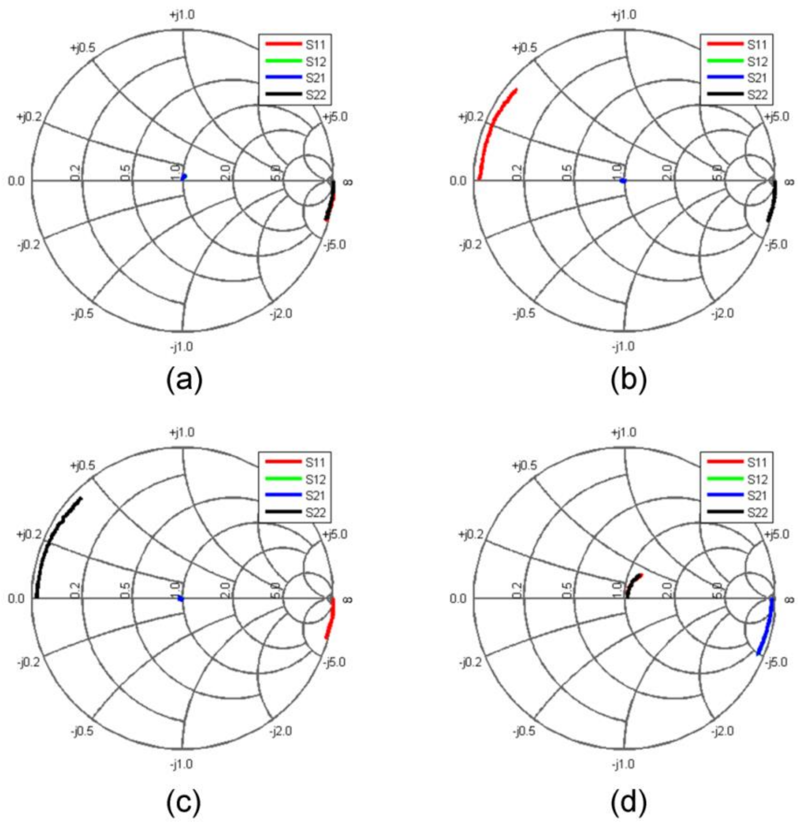

Figure S4. S-parameters of the de-embedding structures. (a) Open. (b) Short1. (c) Short2. (d) Thru.

Calculation of the Small-Signal Model Components:

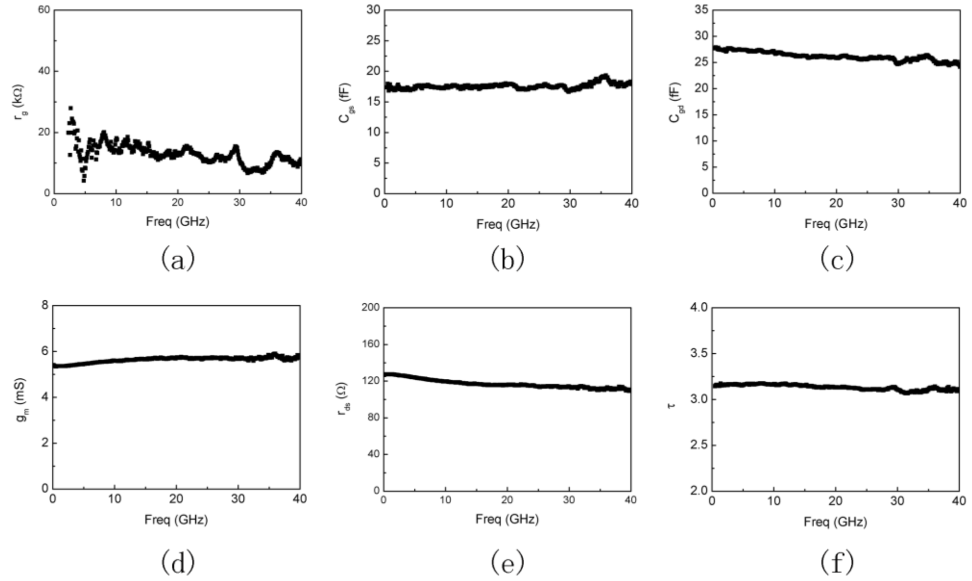

Figure S5. Results of the small-signal model components calculated from S-parameter measurement. (a) r_g. (b) C_{gs}. (c) C_{gd}. (d) g_m. (e) r_{ds}. (f) τ. The parameter extraction is based on Y-parameters calculated from the original S-parameters. The results are very stable, confirming the effectiveness of the small-signal model. We use the average values for the model components, as displayed in Table 1.

h_{21} and MUG of the 300- and 200-nm-gate-length GFETs before De-embedding:

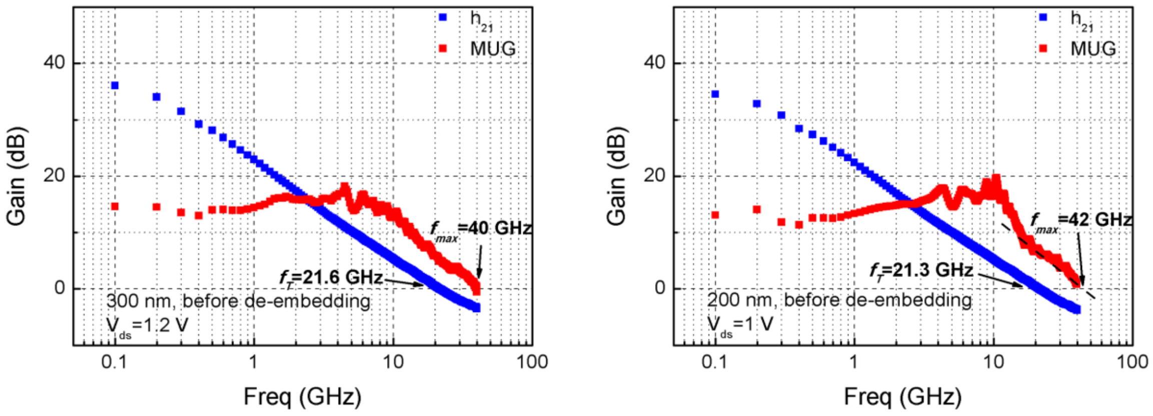

(a) (b)

Figure S6. h_{21} and MUG of the 300- and 200-nm-gate-length GFETs before de-embedding. f_T/f_{max} equal 21.6/40 GHz and 21.3/42 GHz before de-embedding for the 300- (a) and 200- (b) nm-gate-length GFETs, respectively.

***f_T*'s Dependence on Gate Length:**

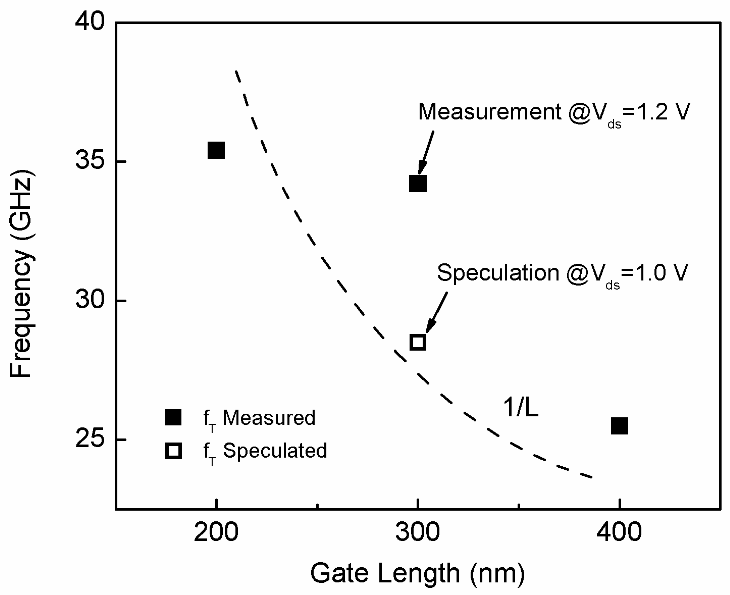

Figure S7. f_T of the 200-, 300- and 400-nm-gate-length GFETs. Note that the 200 nm and 400 nm GFETs were measured with V_{ds}=1.0 V bias, while the 300 nm GFET was measured with V_{ds}=1.2 V bias. As theoretically f_T should be proportional to V_{ds}, (GFETs work as MOSFETs in linear region and transconductance is proportional to V_{ds}) we speculate the f_T of the 300 nm GFET to be around 28. 5 GHz. f_T is negatively dependent on gate length and the relationship is near to 1/L. Limited discrepancy may result from source/drain contact resistances, which play an unignorable role in GFETs.
